# Step-Wise Assembly of LAT Signaling Clusters Immediately After T Cell Receptor Triggering Contributes to Signal Propagation

**DOI:** 10.3390/ijms26094076

**Published:** 2025-04-25

**Authors:** Jieqiong Lou, Elvis Pandžić, Till Böcking, Qiji Deng, Jérémie Rossy, Katharina Gaus

**Affiliations:** 1 EMBL Australia Node in Single Molecule Science, School of Biomedical Sciences, University of New South Wales, Sydney, NSW 2052, Australia; 2 ARC Centre of Excellence in Advanced Molecular Imaging, University of New South Wales, Sydney, NSW 2052, Australia; 3 School of Physics, University of Melbourne, Melbourne, VIC 3010, Australia; 4 Katharina Gaus Light Microscopy Facility, Mark Wainwright Analytical Centre, University of New South Wales, Sydney, NSW 2052, Australia; 5 Biotechnology Institute Thurgau, University of Konstanz, 78464 Konstanz, Germany

**Keywords:** T cell receptor signaling, LAT, single molecule imaging, spatial organization, nanoclusters, plasma membrane, signalosome, protein assembly, signal transduction

## Abstract

Linker for activation of T cells (LAT) is an essential adaptor protein in early T cell receptor (TCR) signaling that propagates multiple signaling pathways. However, how LAT spatial organization facilitates signal initiation and propagation after TCR triggering is not clear. To differentiate de novo assembly in the plasma membrane from pre-existing LAT vesicles and clusters, we developed imaging protocols and analyses to capture the organization and dynamics of single LAT molecules immediately after TCR engagement. We could observe individual LAT molecules in the plasma membrane that assembled into immobile signaling entities requiring LAT phosphorylation. This step-wise assembly process was temporally highly coordinated via the zeta-chain-associated protein kinase 70 (Zap70)-LAT-growth factor receptor-bound protein 2 (Grb2) pathway. While multiple spatial organization co-existed even within the plasma membrane, our data suggest that de novo plasma membrane assemblies facilitated signal propagation.

## 1. Introduction

T cell activation is an essential process in adaptive immunity. Antigen recognition by the T cell receptor (TCR) initiates intracellular signaling that drives T cell activation and fate decisions. Since the first description of the immunological synapse [1,2], the spatiotemporal organization of signaling proteins has been recognized as playing a role in proofreading TCR triggering, resulting in digital activation outcomes [3].

T cell signaling begins with the phosphorylation of the TCR-CD3 complex by the kinase Lck (lymphocyte-specific protein tyrosine kinase), which enables the recruitment and phosphorylation of ZAP70 (zeta-chain-associated protein kinase 70). ZAP70 in turn phosphorylates the adaptor protein LAT (linker for activation of T cells) that functions as a signaling hub [4,5,6,7]. Genetic deletion of LAT or mutation of its tyrosine residues impairs T cell activation and leads to immune deficiencies and autoimmunity [8,9,10,11,12]. LAT is a transmembrane protein with a short extracellular domain and a flexible cytosolic tail. Phosphorylated tyrosine residues in the cytosolic tail serve as docking sites for multiple downstream signaling and adaptor proteins such as Grb2 (growth factor-bound protein), Gads (Grb2 related adaptor downstream of Shc), PLC-γ1 (phospholipase C gamma 1) and SOS1 (Son of Sevenless homolog1) [7,13].

Using advanced proteomics, Malissen and colleagues mapped the LAT interactome, identifying 112 binding partners, and proposed that the so-called LAT signalosome facilitates amplification and diversification of TCR signals [10]. This raises the question of how the molecular events involving LAT are organized in time and space to achieve functionally distinct outcomes. It is already known that LAT exists in two distinct pools in T cells [14]: in the plasma membrane and in intracellular vesicles. LAT in the plasma membrane forms clusters at the immunological synapse immediately after TCR triggering [15,16], while the vesicular pool of LAT is delivered to the immunological synapse from intracellular compartments a few minutes after T cell activation [17,18,19]. There is strong evidence that both LAT surface clusters and LAT vesicles participate in TCR signaling and are required for T cell activation [4,15,17,20]. It is thus possible that the same molecule—LAT—creates different signaling protein networks depending on its subcellular location.

LAT surface clusters, ranging in size from 70–300 nm, were first described by transmission electron microscopy [21] and later confirmed with single molecule localization microscopy (SMLM) [18,22,23]. In diffraction-limited images, LAT and TCR appear to co-localize within microclusters [16], but subsequent SMLM studies proposed that LAT and TCR cluster coalesced [22] or partially overlapped [23]. While it was initially thought that LAT clustering in the plasma membrane was mediated by lipid rafts via the two palmitoylated cysteine residues in LAT, it now appears that palmitoylated-deficient LAT was poorly expressed at the cell surface and LAT phosphorylation was raft independent [24]. Similarly, single particle tracking revealed that LAT trapping in clusters required LAT phosphorylation but not LAT palmitoylation [25]. Alternative mechanisms of LAT clustering could involve downstream adaptors such as Grb2 and SOS1 essentially acting as scaffolds [13]. Indeed, reconstituting phosphorylated LAT (pLAT), Grb2 and SOS in supported lipid bilayers resulted in protein phase separation and dephosphorylation of lead caused the clusters to disassemble [26]. Other reconstitution studies proposed that the lifetime of LAT-Grb2-SOS1 assemblies could be an element of the “kinetic proofreading” that determines the probability of TCR triggering in response to an antigenic peptide [27,28]. In summary, recent evidence in the literature suggests that the spatial organization of LAT is crucial in determining the initiation and progression of T cell activation”.

Because a molecular understanding of the assembly of LAT clusters may also provide fundamental insights into signal propagation, we focused on the first events after TCR engagement, before LAT from intracellular pools comes into play, combining imaging techniques such as step fitting analysis of live-cell total internal reflection fluorescent (TIRF) images [29,30], membrane diffusion analysis with k-space imaging correlation spectroscopy (kICS) analysis [31,32] and SMLM cluster and co-localization (DoC) analysis [33]. Our results show that LAT clusters assemble molecule by molecule in the plasma membrane which requires tyrosine phosphorylation of LAT. The step-wise assembly of the LAT signaling platform at the onset of TCR activation could act as a kinetic limiting factor that determines the probability of TCR triggering in response to an antigenic peptide.

## 2. Results

### 2.1. Imaging LAT Assembling in the Plasma Membrane Revealed a Step-Wise Assembly Process Immediately After TCR Triggering

To test whether TIRF microscopy could be used to distinguish between plasma membrane LAT and vesicular LAT during the early TCR activation phase (Figure S1), we made a LAT construct that is the anti-CD4 antibody only accessible to plasma membrane LAT molecules. Specifically, this construct consists of LAT fused to mCherry on the intracellular side and to the extracellular domain of CD4 [15] (Figure S1A). We acquired live-cell TIRF movies of LAT-deficient T cells expressing CD4-LAT-mCherry on activating surfaces after cells were labelled on ice with anti-CD4 antibodies conjugated to AlexaFluor 488 (Figure S1B). In the earliest detectable LAT assemblies (<2 min), the mCherry signal colocalized with the anti-CD4 AlexaFluor 488 signal, indicating that these assemblies resided exclusively in the plasma membrane (Figure S1B, Movie S1). Antibody inaccessible LAT, characterized by red-only fluorescence, appeared later (>2 min Figure S1B,C), suggesting that vesicular LAT arrives much later at the activation site, as previously suggested [14,18]. It was noticeable that the antibody-stained LAT assemblies in the plasma membrane (bright yellow puncta in Figure S1) were largely immobile, persisted over the entire imaging period (>10 min) and were mainly found at the periphery of the activation zone, while LAT vesicles were more transient and appeared in the central activation zone (Movie S1). We concluded that LAT signals in the first 2 min of T cell activation under TIRF illumination arose predominantly from plasma membrane. We therefore used LAT fusion proteins without the CD4 domain for subsequent experiments.

**Figure 1:**
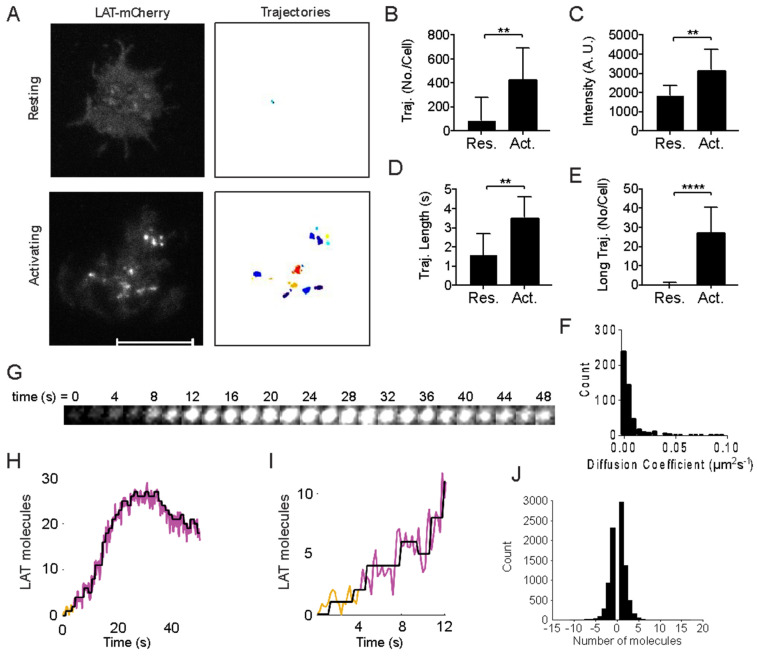
LAT assembled step-wise into immobile entities in the plasma membrane in activating but not resting T cells. LAT-deficient Jurkat cells reconstituted with LAT-mCherry were exposed to non-activating surfaces (poly-L-lysine-coated coverslips, resting) and activating surfaces (anti-CD3 and anti-CD28 antibodies coated coverslips, activating). TIRF images of individual cells were recorded at a high frame rate (5 frames/s) before and up to 2 min after cells made contact with the coverslips. (**A**) TIRF images (left) and identified trajectories (right) of LAT-mCherry in resting (top) and activating (bottom) T cells. Scale bar = 10 µm. (**B**–**E**) Mean number of trajectories per cell (**B**), total intensity per assembly (**C**), and trajectory length (**D**) of all identified trajectories with ≥2 frame (≥400 ms) and number of trajectories per cell that spanned ≥ 6 s (≥30 frames) (**E**) in resting and activating T cells. Data are mean and standard deviation from *n* = 3 independent experiments with at least 8 image series per condition; ** *p* < 0.01, **** *p* < 0.0001 (unpaired *t*-test). (**F**) Distribution of diffusion coefficients extracted from trajectories tracked over ≥30 frames (≥6 s) in activated T cells. (**G**–**I**) Example of a LAT-mCherry trajectory in a T cell on an activating surface and the corresponding step-fitting analysis over the entire trajectory (**H**) and the first 60 frames (12 s, (**I**) of the example shown in (**G**)). Trajectories were extended back in time (20 frames, yellow) to capture the initial assembly phase. (**J**) Number of LAT-mCherry molecules that joined and dissociated from LAT trajectories during the first 2 min of activation. Data were derived from *n* = 535 trajectories spanning ≥30 frames (≥6 s).

We next visualized LAT assembly in the plasma membrane by imaging LAT-mCherry expressed in LAT-deficient cells immediately after cells made contact with the coverslip, acquiring 300 frames (1 min in total) with a high acquisition rate (5 frames/s) and low laser intensity for minimizing photobleaching (Figure S1A). To achieve this, glass surfaces were coated with poly-L-lysine or with anti-CD3 and anti-CD28 antibodies to image resting T cells and activating T cells, respectively. In resting cells, bright LAT assemblies were rarely visible (Figure 1A, Movie S2). In contrast, in activating T cells, bright LAT assemblies quickly assembled during the cell-spreading phase, persisted over long periods and gained brightness over time (Figure 1A, Movie S3).

We used a single-particle tracking (SPT) algorithm to identify individual LAT trajectories and quantify their total fluorescence intensity over time. The algorithm identified the center of bright LAT assemblies as regions where the local pixel mean intensity (within a 6-pixel diameter) was at least twice the plasma membrane LAT mean intensity. Assemblies were then tracked across frames if their center shifted by no more than 3 pixels in the x and y directions between consecutive frames, with no gap closing allowed. LAT trajectories were defined as assemblies that remained detectable for at least two consecutive frames. We observed significantly more LAT trajectories during the first minute of cell contact with activating surfaces than with non-activating/resting surfaces (Figure 1B). LAT assemblies in activating cells were both brighter (Figure 1C) and lasted longer (Figure 1D) than those in resting cells. Notably, LAT trajectories lasting ≥6 s (≥30 frames) were rare in resting T cells (Figure 1E), whereas long-lived LAT trajectories in activating T cells were frequent and largely immobile (Figure 1F). Together, these results suggest that stable and immobile LAT assemblies form in the plasma membrane immediately after TCR triggering. This aligns with previous findings that TCR activation and LAT phosphorylation promote the formation of large-scale LAT clusters in the plasma membrane [13,26,28,34].

To gain insights into whether LAT assemblies post TCR triggering were assembled molecule by molecule or ‘in bulk’, we applied a step-fitting algorithm to the long-lived and immobile (<0.05 µm^2^/s) trajectories in activated T cells (Figure 1G–I) [29]. We calibrated the fluorescence emitted from single mCherry molecules by imaging purified mCherry absorbed on cleaned cover glass under identical imaging conditions as in the live cell LAT-mCherry experiments (Figure S2B). Single mCherry was identified by two criteria: (1) the mCherry molecule was identified in the first frame, and (2) single step photo-bleaching happened during the movie acquisition (Figure S2C). The mean intensity difference before and after single-step photobleaching was calculated as single mCherry intensity (Figure S2C). The mean intensity of single mCherry was 111 ± 40 standard deviation in our imaging condition (Figure S2D). To ensure that we captured the very beginning of the LAT assembling process, we included 20 frames before the detection of the LAT assembly. As can be seen in Figure 1H,I (more examples are shown in Figure S2E), LAT assemblies started with a single LAT molecule and grew molecule by molecule. After ~35 s, we also observed individual molecules dissociating from the assemblies but without complete disassembly of the LAT entity in the imaging time frame (1 min in total). In conclusion, the step-fitting analysis revealed that the de novo LAT assemblies were initiated by adding one or two LAT molecules in response to TCR activation (Figure 1J).

### 2.2. LAT Molecules Diffused Slower and Are More Confined After TCR Activation

**Figure 2:**
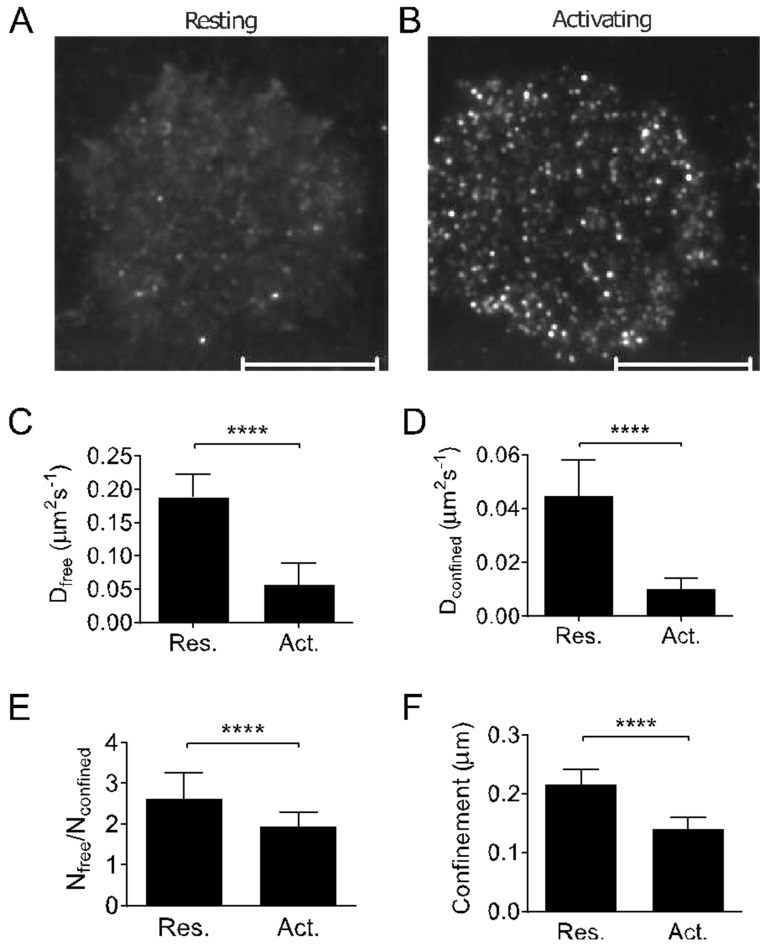
Diffusion and confinement of LAT molecules in the plasma membrane of resting and activating T cells as revealed by kICS. LAT-deficient Jurkat cells expressing LAT-mEOS2 were exposed to non-activating surfaces (poly-L-lysine coated coverslips, resting) and surfaces activating (anti-CD3 and anti-CD28 antibodies coated coverslips, activating) for 0–5 min. TIRF images series underwent kICS analysis and spatiotemporal correlation functions fitted to two populations of LAT molecules diffusing on ‘free’ and ‘confined’ spatial scales, as described in Figure S3. (**A**,**B**) Merged TIRF images of 3000 frames (mean intensity) of a resting T cell (**A**) and activating T cells (**B**). Scale bar = 10 µm. (**C**,**D**) Diffusion coefficients of the ‘free’ (**C**) and ‘confined’ (**D**) populations. (**E**) Ratio of LAT molecules in the ‘free’ vs. ‘confined’ population. (**F**) Length scale of the ‘confined’ population. In (**C**–**F**), data are means and standard deviations from ≥15 cells and from ≥3 independent experiments. **** *p* < 0.0001 (unpaired *t*-test).

To understand how LAT molecules diffuse and become trapped in assemblies within the plasma membrane, we performed k-space image correlation spectroscopy (kICS) [32] of LAT-mEOS2 in live T cells on non-activating and activating surfaces under TIRF illumination (Figure 2A,B, Movie S4). We used an extension version of kICS that can distinguish between diffusing and confined proteins within the plasma membrane [31,35,36]. In brief, fluorescence fluctuation correlations between all pixel pairs in an image time series are calculated, and pairs with the same spatial and temporal lags are grouped to produce a spatiotemporal correlation function (Figure S3A,B). By fitting the correlation function, we extracted the dynamics of the dominant LAT populations in the plasma membrane. Photo-activation of LAT-mEOS2 ensured that individual LAT molecules were recorded that diffused within the plasma membrane. Fluorescence fluctuations arising from molecules entering and leaving the TIRF zone (i.e., vesicles) only influence the amplitude of the correlation function at zero spatial lag, not at any other spatial frequencies [37].

The spatiotemporal correlation function of LAT-mEOS2 did not exhibit a mono-exponential decay (Figure S3B). This means that at least two populations of diffusing LAT species existed and gave rise to correlations that differ in the spatial scale on which they diffuse [31,35,36]. Diffusion on the large spatial scale, which we refer to as the ‘freely diffusing’ population, took place outside LAT assemblies. Diffusion on the small and confined spatial scale, which we will refer to as the ‘confined’ population, took place within LAT assemblies. By fitting the correlation functions for every lag time for the ‘freely diffusing’ and ‘confined’ populations (Figure S3B–D), we obtained the diffusion coefficients Dfree and Dconfined (Figure 2C,D), and the relative distribution of LAT molecules between the two populations (Figure 2E and Figure S3E) as well as the spatial length scale on which ‘confined’ population experience confinement (Figure 2F and Figure S3D). The last parameter has been shown in simulation to depend on the sizes of domains and the confinement trapping strength. For very strong trapping strength as experienced by LAT molecules during the TCR triggering, individual LAT molecules will explore smaller confinement lengths, as shown by the decrease in confinement length scale (Figure 2F).

TCR triggering reduced the diffusion coefficients of both ‘freely diffusing’ and ‘confined’ LAT populations (Figure 2C,D), suggesting that T cell activation results in a global membrane organization that impacts LAT diffusion. We also observed a decrease in the ratio of freely diffusing LAT molecules versus LAT molecules that exhibited confined diffusion within assemblies (Figure 2E) and a shortening of the confinement length scale (Figure 2F). This suggests that LAT assemblies in activated T cells were denser and retained LAT molecules more strongly than LAT assemblies in resting T cells. Thus, the kICS analysis confirmed the co-existence of at least two LAT populations within the plasma membrane, suggesting a constant exchange of LAT molecules between clusters and the rest of the plasma membrane. They further suggest that the properties of the plasma membrane of activated T cells shift the equilibrium towards less diffusion and more clustering of LAT molecules.

### 2.3. SMLM Revealed Diversity in LAT Clusters in the Plasma Membrane

**Figure 3:**
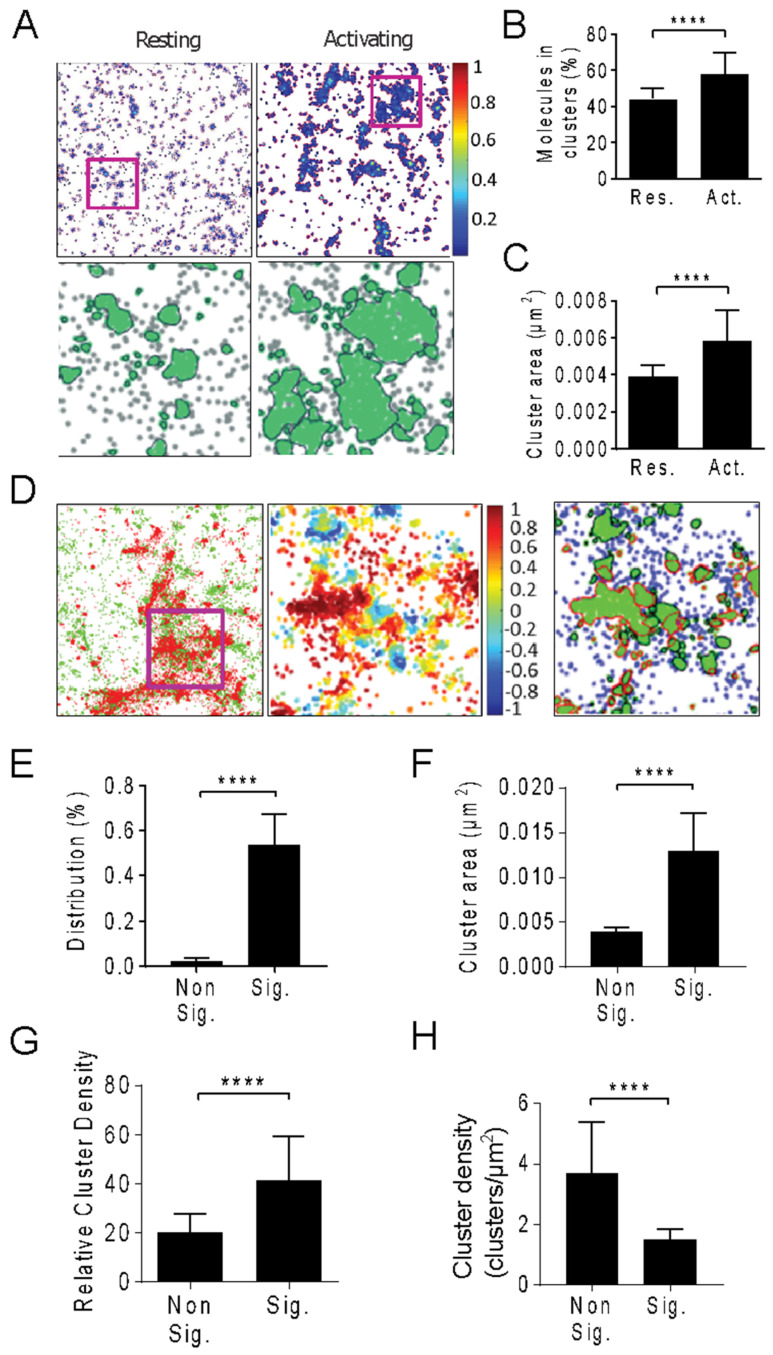
Identification of LAT clusters and phosphorylated LAT clusters after 2 min of activation with SMLM. LAT-deficient Jurkat cells expressing LAT-mEOS2 were exposed to non-activating surfaces (poly-L-lysine coated coverslips, resting) and activating surfaces (anti-CD3 and anti-CD28 antibodies coated coverslips, activating) for 2 min, fixed and stained for phosphorylated LAT (pLAT_191_). (**A**) SMLM images of LAT-mEOS2 in resting and 2-min-activating Jurkat cells. Pseudo-colored SMLM images are displayed based on normalized molecular density within a 4 μm × 4 μm region. Cluster analysis of the region of interest (1 μm × 1 μm), highlighted by a magenta square in A (top), shows LAT molecules in clusters (green) and molecules outside clusters (gray). The outlines of the LAT clusters are traced with black lines. (**B**,**C**). Quantification of percentage of molecule in cluster (**B**) and cluster area (**C**) in resting and activating Jurkat cells *(n* ≥ 45 ROIs from at least 10 cells in 3 independent experiments). (**D**) SMLM image regions (4 μm × 4 μm) of LAT-mEOS2 (green) and pLAT (red, (**D**) left), and the degree of co-localization (DoC) map ((**D**) middle) and thresholded DoC map ((**D**) right) of the magenta square highlighted region (magenta box, 1.5 μm × 1.5 μm). DoC scores, indicated by color scale, range from −1 to 1, with −1 indicating segregation, 0 indicating no association and +1 indicating colocalization. The thresholded DoC map shows LAT molecules outside clusters (blue), molecules in signaling clusters (green dots, red outline) and non-signaling clusters (green dots, black outline). (**E**). The percentage of signaling LAT molecules resides in non-signaling and signaling LAT clusters. (**F**–**H)** Cluster size (**F**), Relative molecular density in clusters (**G**), and number of clusters per μm^2^ (**H**) of non-signaling (Non Sig.) and signaling (Sig.) clusters (*n* ≥ 39 ROIs from at least 11 cells in 4 independent experiments). In (**B**,**C**,**E**–**H**), data are means and standard deviations per condition. **** *p* < 0.0001 (unpaired *t*-test).

Single molecule step-fitting analysis and image correlation analysis both point towards the molecular assembly of large and immobile LAT clusters, with the LAT molecule diffusion coefficient dropping below 0.02 µm^2^/s upon TCR triggering (Figure 2D). Next, we assessed LAT clustering with SMLM imaging in activating and resting T cells (Figure 3A) and investigated the distribution of phosphorylated LAT (pLAT) relative to LAT clusters with two-color SMLM (Figure 3D). We imaged LAT via the fluorescent fusion protein mEOS2 and pLAT with an Alexa Fluorophore 647 (AF647) tagged pTyr220-specific antibody [38]. We grouped re-excited molecules as previously described (Annibale et al., 2011) [39] and achieved a localization precision of 25.1 ± 7.7 nm for LAT-mEOS2 and 15.5 ± 6.3 nm for pLAT (Figure S4A). We quantified LAT clusters with an algorithm called density-based spatial clustering of application with noise (DBSCAN) that connects individual molecules in a propagative manner if they have ≥3 neighbors in a 20-nm radius. As observed previously with different cluster analyses [3,4], activated T cells had more LAT molecules in clusters (Figure 3B) and larger average cluster sizes (Figure 3C) than resting T cells. A rough estimate of the number of LAT molecules per cluster measured with SMLM (34 ± 24, Figure S4B) agreed with the step-fitting analysis (28 ± 5, Figure 1H and Figure S2E). The overall density of LAT molecules detected in the TIRF zone did not change after 2 min of activation, strongly suggesting that, at this early time point, LAT clustering was caused by the reorganization of LAT molecules already in the plasma membrane [15]. In contrast, we and others previously reported that vesicular LAT was recruited to the T cell activation site at ~10 min post TCR triggering (Figure S1), resulting in a greater LAT molecule density (Figure S4C) [17,18,22]. Taken together, the data suggest that there were two distinct phases of LAT reorganization: in the initial phase (<2 min), LAT clusters were formed by assembling individual LAT molecules in the plasma membrane (Figure 1 and Figure 2), which was followed by recruitment of vesicular LAT at a later stage (~10 min) [14,17,18,22,23].

Next, we distinguished signaling from non-signaling LAT molecules with a degree-of-colocalization (DoC, Figure 3D) analysis as previously described [33]. This is necessary because two labels on the same protein do not completely spatially overlap in dual-color SMLM images. DoC analysis correlates density gradients of LAT with density gradients of pLAT for each LAT molecule (calculated by counting LAT and pLAT molecules in concentering rings of increasing radii around each LAT molecule) and assigns a DoC value to each LAT molecule ranging from +1 (colocalization) to −1 (segregation). We regarded a LAT molecule as a signaling LAT molecule if its DoC score was ≥ 0.4 (Figure 3D). In addition, we used DBSCAN analysis to identify LAT clusters, as shown in Figure 3A. Clusters containing at least 10 signaling LAT molecules (DoC score ≥ 0.4) were classified as signaling clusters, while the rest were considered non-signaling clusters (Figure 3D).By performing this two-step analysis, we noticed that a significant proportion of signaling LAT molecules (~44%) resided outside clusters, indicating that LAT phosphorylation may not necessarily occur within clusters. In agreement with the single particle tracking results (Figure 1), we discovered that signaling and non-signaling LAT clusters are significantly different: signaling clusters were larger (Figure 3F) and denser (Figure 3G) but were less frequent than non-signaling clusters (Figure 3H). This suggests that LAT clustering may occur through two distinct mechanisms: one involving large, dense clusters linked to LAT phosphorylation, and another where LAT molecules associate with LAT phosphorylation, such as in resting T cells. These different types of LAT clusters could explain discrepancies in SMLM data regarding the co-localization of LAT and TCR clusters [22,40].

### 2.4. Phosphorylation Is Required for LAT Assembly in the Plasma Membrane

Given that one class of LAT clusters were phosphorylated, we investigated whether LAT phosphorylation was required for the step-wise LAT assembly process in the plasma membrane seen in Figure 1. Thus, we expressed in LAT-deficient cells a LAT signaling mutant (LATYF), in which all nine tyrosine in the cytosolic tail were mutated to phenylalanine.

**Figure 4:**
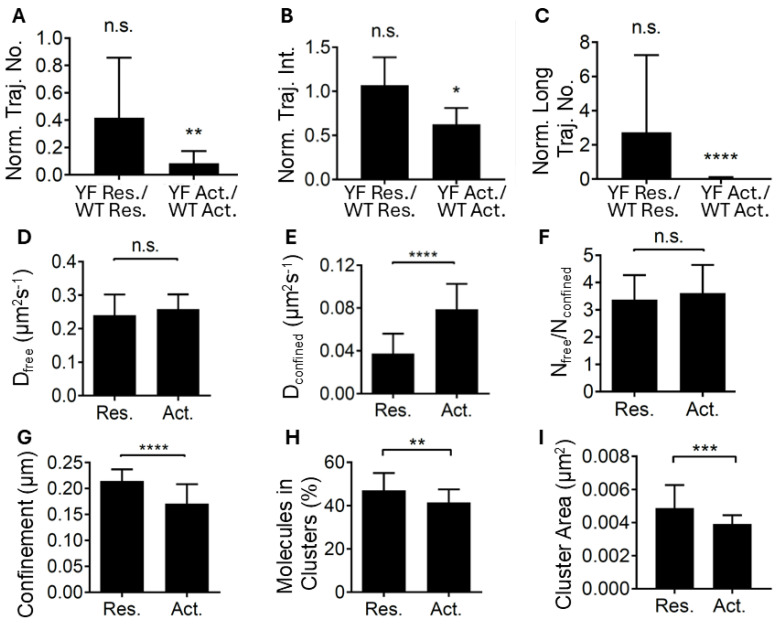
Tyrosine phosphorylation is required for LAT assembly in the plasma membrane in the early phase of T cell activation. LAT-deficient Jurkat cells expressing LAT_YF_-mCherry (**A**–**C**) or LAT_YF_-mEOS2 (**D**–**I**) were exposed to non-activating surfaces (poly-L-lysine coated coverslips, Res.) and activating surfaces (anti-CD3 and anti-CD28 antibodies, Act.) for 2 min. (**A**–**C**) SPT imaging and analysis were performed as described in Figure 1. The number of LATYF-mCherry trajectories (≥2 frames, 400 ms, (**A**)), intensity of all identified LATYF-mCherry assemblies (**B**) and the number of long-lived LATYF-mCherry trajectories (≥30 frames, 6 s, (**C**)) was normalized to wild-type LAT-mCherry trajectories in resting (WT Res.) or activating conditions (WT Act.). Data are means and standard deviations of ≥11 cells per condition. n.s. *p* > 0.05, * *p* < 0.05, ** *p* < 0.01, **** *p* < 0.0001 (unpaired *t*-test between LATYF Res. vs. WT LAT Res. and LATYF Act. vs. WT LAT Act.). (**D**–**G**) kICS analysis was performed as described in Figure 2. Diffusion coefficients of the ‘free’ (**D**) and ‘confined’ (**E**) populations of LATYF-mEOS2. Ratio of LATYF molecules in the ‘free’ vs. ‘confined’ population (**F**) and length scale of the ‘confined’ population (**G**). Data are means and standard deviations from ≥31 cells from 4 independent experiments. n.s. *p* > 0.05, * *p* < 0.05, ** *p* < 0.01, **** *p* < 0.0001 (unpaired *t*-test). (**H**,**I**) SMLM imaging of LATYF-mEOS2 and cluster analysis was performed as in Figure 3 to extract the percentage of molecule in cluster (**H**) and cluster size (**I**). Data are means and standard deviations of ≥10 cells per condition from 3 independent experiments. ** *p* < 0.01, *** *p* < 0.001 (unpaired *t*-test).

While T cells expressing LATYF-mCherry can spread on activating surfaces (Movie S5), they do not form as many stable and bright assemblies as wild-type LAT-mCherry (Figure 1, Movie S3). However, resting cells expressing LATYF show a similar number of LAT entities compared to wild-type LAT. To directly compare whether LATYF is required for LAT assembly formation in response to TCR activation, we normalized the number and intensity of LATYF assemblies in both resting and activating conditions to those of wild-type LAT under corresponding conditions. We found a significantly lower number of LATYF assemblies than wild-type assemblies in activated cells (Figure 4A). Similarly, in activated cells, LATYF assemblies cells were less bright than wild-type LAT assemblies and were indistinguishable from wild-type LAT trajectories in resting cells (Figure 4B). We could not apply the step-fitting analysis to LATYF assemblies because almost none of them had a sufficiently long lifetime (Figure 4C). Taken together, these data show that LATYF did not assemble into stable clusters upon TCR triggering, suggesting that phosphorylation of LAT was a prerequisite for the de novo formation of large and immobile clusters in the plasma membrane. It is noteworthy that spontaneous clustering events could still be observed with LATYF, similar to wild-type LAT in resting T cells, and may lead to the non-signaling clusters observed with SMLM. The recruitment of vesicular LAT at ~10 min post activation also did not require LAT phosphorylation [18].

We then repeated the kICS analysis with LATYF-mEOS2 to determine whether LAT phosphorylation plays a role in the decrease in LAT diffusion that we observed for wild-type LAT upon TCR activation. Similar to wild-type LAT, LATYF had two diffusive populations (Movie S6). However, unlike wild-type LAT, the ‘free’ diffusion coefficient, representing diffusion of LAT molecules outside clusters, was not modified by TCR activation (Figure 4D), demonstrating that LAT phosphorylation was required for the reduced diffusion caused by TCR triggering. In contrast to wild-type LAT (Figure 2D), the ‘confined’ population of LATYF diffused faster in activating cells than in resting cells (Figure 4E), suggesting that LATYF clusters failed to become denser upon TCR activation. The absence of an assembly process in activating T cells expressing LATYF was also evident, in that TCR triggering did not redistribute LATYF molecules diffusing inside and outside of clusters (Figure 4F). Interestingly, the length scale on which confinement was experienced by LAT molecules was reduced in T cells expressing either wild-type LAT (Figure 2F) or LATYF (Figure 4G). Taken together with the decrease of diffusion coefficient at all spatial scales for wild-type LAT compared to the increase of diffusion coefficient at small spatial scales for LATYF, this indicates that T cell activation caused local membrane reorganization, which affected the diffusion on small spatial scales of plasma membrane LAT independently of phosphorylation status.

SMLM imaging of LATYF–mEOS2 and DBCAN analysis revealed that, similarly to wild-type LAT, LATYF clusters existed in both resting and activating cells. However, TCR activation did not increase, but decreased the percentage of LATYF molecules in clusters (Figure 4H vs. Figure 3B for wild-type LAT), the size of the clusters (Figure 4I vs. Figure 3C) and the number of molecules per cluster (Figure S5C vs. Figure S4C). Interestingly, a point-mutation in the 6th tyrosine of LAT, LATY6F, exhibited similar diffusion and clustering behavior as wild-type LAT upon TCR triggering (Figure S6), suggesting that full LAT phosphorylation is not required for cluster assembly. In conclusion, the three lines of evidence—detection of molecular assemblies in live cells with single particle tracking, diffusion with kICS and clustering measured by SMLM—demonstrate that phosphorylation of LAT was required for the formation of large and immobile LAT clusters in the plasma membrane during the first 2 min of TCR activation.

### 2.5. LAT Assemblies Formed in a Highly Coordinated Fashion and Propagated TCR Signaling via the ZAP70-LAT-Grb2 Pathway

**Figure 5:**
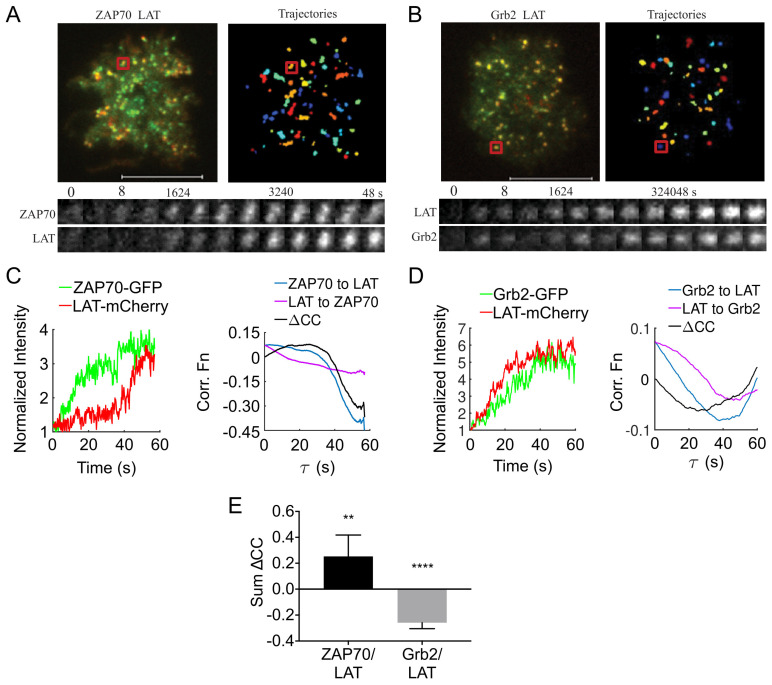
De novo LAT membrane assemblies signal via the Zap70-LAT-Grb2 pathway. LAT-deficient Jurkat cells expressing ZAP70-GFP and LAT-mCherry or Grb2-GFP and LAT-mCherry were exposed to activating surfaces (anti-CD3 and anti-CD28 antibodies coated coverslips). TIRF images of individual cells were recorded at high frame rates (5 frames/s) before and up to 2 min after cells made contact with the coverslips. (**A**,**B**) Left, merged TIRF images of ZAP70-GFP (green) and LAT-mCherry (red) (**A**); Grb2-GFP (green) and LAT-mCherry (red) (**B**). Right, identified ZAP70-GFP (**A**) or LAT-mCherry (**B**) trajectories. Scale bar = 10 µm. Image time series shown in A and B examplify trajectory intensity montage of the red square highlighted regions in (**A**,**B**). (**C**,**D**) Left: Intensity profile (normalized to minimum intensity in each channel) of a LAT (red) and ZAP70 (green) trajectory (**C**) and a LAT (red) and Grb2 (green) trajectory (**D**). Right: The cross-correlation plot for correlations from ZAP70-GFP or Grb2-GFP to LAT-mCherry (blue line) and from LAT-mCherry to ZAP70-GFP or Grb2-GFP (purple line) and the difference between the two cross-correlation functions (ΔCC, black line) (**E**) Mean differences of the cross-correlation functions (ΔCC) for the first 8.3 s lag. Positive values for the correlation between LAT and ZAP70 indicate that ZAP70 preceded the LAT assembly and inversely, negative values for the correlation between LAT and Grb2 indicate that Grb2 recruitment followed LAT assembly. Data are mean and 95% confidence interval from 210 trajectories and 14 cells (ZAP70-GFP and LAT-mCherry) and 308 trajectories and 14 cells (Grb2-GFP and LAT-mCherry), respectively. ** *p* < 0.01, **** *p* < 0.0001, one-sample *t*-test compared to hypothetical value zero.

Since LAT phosphorylation was essential for the assembly of the LAT signalosome in the plasma membrane, we examined the temporal sequence between LAT clustering and the recruitment of the main upstream kinase, ZAP70 [7], and the main downstream adaptor protein, Grb2, which binds to multiple phosphorylated tyrosines on the LAT cytosolic tail [13]. We used two-color TIRF imaging with acquisition rates (5 frame/s) to simultaneously record LAT-mCherry and ZAP70-GFP or LAT-mCherry and Grb2-GFP in the first 2 min of cells contacting the activating surface (Figure 5A,B). In the resulting movies, it is notable that the formation of ZAP70 puncta preceded the assembly of LAT clusters (Figure 5A image time series Movie S7). In contrast, Grb2 accumulated at a similar time to LAT clustering (Figure 5B image time series, Movie S8). 

To quantify the movies, we normalized the intensity to the minimum intensity of long-lived (≥6 s) two-color punctate that were almost immobile (diffusion coefficient ≤0.05 μm^2^/s) and applied a temporal cross-correlation function. In the temporal cross-correlation function, the intensities of the reference channel—either ZAP70-GFP (Figure 5C, left) or Grb2-GFP (Figure 5D, left)—were correlated with the intensity of LAT-mCherry. A peak in the cross-correlation function indicates the time delay (τ) for the appearance of the GFP-tagged protein relative to LAT-mCherry. By reversing the reference channel (i.e., using LAT-mCherry as the reference for correlation with ZAP70-GFP or Grb2-GFP), two temporal cross-correlation curves were generated for each comparison (blue and purple lines, Figure 5C,D right panels).

We then calculated the difference between these two cross-correlation curves (ΔCC, black lines in Figure 5C,D, right panels) across all values of τ. In the resulting ΔCC curves, positive values indicate that the GFP-tagged protein was recruited to the forming clusters before LAT-mCherry. Conversely, negative values signify that LAT-mCherry clustering preceded the recruitment of the GFP fusion protein. Zero values across all temporal lags (τ) suggest that the recruitment of the two species occurred independently, with no temporal ordering between them [41].

In the examples shown in Figure 5, we found a positive cross-correlation for short lag times between ZAP70-GFP and LAT-mCherry (ΔCC, black line in Figure 5C) indicating that ZAP70 assembly preceded LAT assembly. In contrast, we found a negative cross-correlation between Grb2-GFP and LAT-mCherry (ΔCC, black line in Figure 5D), suggesting that GRB2 followed LAT assembly. Since cross-correlation curves can be noisy, we binned the values for τ = 0–8.3 s (sum ΔCC = sum of first 50-time lags of ΔCC) and calculated the mean for many two-color puncta (Figure 5E). The mean sum ΔCC value was significantly greater than zero for ZAP70-GFP and LAT-mCherry and significantly lower than zero for Grb2-GFP and LAT-mCherry (Figure 5E), suggesting that the temporal sequence consists of ZAP70 recruitment, LAT assembly within the plasma membrane, and finally the recruitment of Grb2. This strongly suggests the de novo formed LAT entities propagated TCR signaling via the ZAP70-LAT-Grb2 pathway.

## 3. Discussion

LAT is an essential signaling protein for T cell activation that contributes to signal propagation through phosphorylation of its cytosolic tail [4,10,15,20] and signal diversification through its interaction with at least 112 different proteins [42]. The diversification may arise from different spatial organizations. It is now known that at least two pools of LAT exist [19]: a vesicular pool that delivers LAT to the immunological synapse after TCR signaling is initiated [17,18] and may facilitate signal amplification, and a plasma membrane pool. LAT microclusters in the plasma membrane only become visible in live T cells with diffraction-limited microscopy a few minutes after TCR triggering [15] while 2-dimensional SMLM was found pre-existing LAT clusters. We re-examined the organization of LAT in the plasma membrane with a range of imaging technologies that have single molecule sensitivity. Single particle tracking and a temporal cross-correlation analysis allowed us to evaluate individual LAT assemblies over time, while kICS analysis on TIRF image series and a cluster analysis of SMLM data examine the dynamics and distribution, respectively, of all recorded molecules at the cell surface. The latter methods clearly revealed the existence of at least two different spatial organizations of LAT in the plasma membrane of resting and activated T cells. The kICS analysis provides particularly strong evidence for this conclusion, since LAT vesicles docking at the plasma membrane, which could be misidentified as LAT membrane clusters in SMLM data, do not affect the outcome that at least two diffusing LAT populations co-exist: a slowly diffusing confined population, probably LAT molecules in and around clusters, and a freely diffusing population that diffuse at larger spatial scale between clusters that decreased upon TCR triggering. In addition, the degree-of-colocalization analysis of SMLM data identified two populations of LAT membrane clusters after 2 min of activation: large and dense clusters that contain phosphorylated LAT and small and less dense clusters that do not. Combining the evidence from different imaging technologies, we thus conclude that different spatial organizations of LAT co-exist in the plasma membrane, making the identification of the functional relevance of, for example, LAT clustering more challenging.

We provide several lines of evidence that the LAT assemblies that become detectable in the TIRF zone in the first 2 min upon T cells contacting activating surfaces are predominately associated with the plasma membrane. These are: all detectable LAT was recognized by extracellular antibodies (Figure S1); the total amount of LAT molecules remained constant during this period (Figure S4C); LAT assembled and disassembled mainly in one-molecule steps (Figure 1J); LAT entities were largely immobile for prolonged periods of time (Figure 1); and spatiotemporal image correlation exhibited classic 2-component 2-dimensional diffusion characteristics (Figure 2 and Figure S3B).

We identified one LAT population in the plasma membrane that assembled in a step-wise fashion into large and relatively immobile clusters immediately after TCR triggering. The assembly process required the phosphorylation of LAT and followed the ZAP70 recruitment assembly at ‘hot spots’ in the plasma membrane. Thus, is highly likely that the step-wise LAT assembly process is driven by LAT phosphorylation, resulting in the large and dense phosphorylated LAT clusters seen with SMLM. Those are the same clusters that impede LAT diffusion on small spatial scales (Figure 2D) and enrich its density post triggering (Figure 2E and Figure 3B). In contrast, a non-phosphorylatable LAT mutant did not assemble into clusters nor become immobile post TCR triggering, resembling the behavior of wild-type LAT in resting cells. It is thus tempting to speculate that ZAP70 phosphorylation of LAT is the trigger for this assembly process. Interestingly, a previous study of T-cell triggered by antibody coated nano-particles has reported that the ZAP70 negative cell line still exhibited large TCR and phosopo-TCR clusters surrounding and extending beyond the activation sites [43]. Nevertheless, it is entirely plausible that the vesicular LAT pool played a role in the TCR phosphorylation and spatial triggering while the membrane bound LAT molecules we report in this study engage the TCRs via a ZAP70 dependent mechanism. This would explain how this study observed the TCR phosphorylation spatially further away from the direct sites of TCR triggering, near nano-particles’ activation sites.

Supporting evidence for a de novo assembly of LAT clusters in the plasma membrane after TCR triggering also comes from our SMLM observation that LAT molecules outside clusters could be phosphorylated at these early time points. While we cannot formally exclude the possibility that these LAT molecules were phosphorylated in clusters and then dissociated from these clusters, the more likely explanation is that LAT molecules did not have to reside in clusters to become phosphorylated. This is in stark contrast to the TCR-CD3 complex, where receptors in dense clusters had a higher signaling efficiency than receptors in less dense clusters or non-clustered receptors [33]. The existence of non-clustered, phosphorylated LAT is, however, strong evidence that LAT molecules in the plasma membrane were indeed phosphorylated within 2 min after TCR triggering. At later time points, i.e., 10–15 min after TCR triggering, the plasma membrane pool of phosphorylated LAT may constitute the minority because vesicular LAT was not only recruited to the plasma membrane at these later time points but accounted for the majority of phosphorylated LAT [18].

The de novo assembly process was facilitated by the addition of individual LAT molecules. Despite this, the step-wise assembly process was rather rapid, probably because LAT existed at relatively high concentrations in the plasma membrane and few LAT molecules left the assembly. Mutations of LAT that reduce the plasma membrane pool, such as the removal of the palmitoylated cysteines [24], are probably also interfering with the step-wise assembly process in the plasma membrane. In addition, the recruitment of the adaptor proteins such as Grb2 [6,7,13,40] to the nascent LAT assembly could further enhance the stability of the forming LAT clusters, both by reducing its lateral mobility and by building a protein network within the cluster. As previously reported and discussed, it is likely that LAT–protein interactions, rather than association with lipid domains, facilitate the assembly of signaling entities [25,26,34]. Thus, the step-wise assembly is integrally linked to signal propagation, suggesting that as the LAT assembly grows, so do its interactions, facilitating an essentially linear signal progression that eventually becomes irreversible. Whether these LAT clusters preferentially interact with a specific subset of LAT interactors is an interesting area of further research, for which we have now established the appropriate imaging tools.

In conclusion, we provide evidence that multiple types of spatial organization of LAT co-exist in T cells, possibly contributing to the TCR signal diversification by LAT. One of these types of spatial organization is the step-wise assembly of LAT in the plasma membrane immediately after TCR triggering into large, dense and immobile clusters. This molecular assembly process is distinct from global changes in membrane organization that give rise to membrane domains and domain-mediated protein clustering [44]. The assembly process occurred in a temporally highly coordinated manner, linking it with LAT interactions with upstream and downstream binding partners. The resulting LAT clusters are thus likely to be involved in signal initiation and propagation and are different entities to pre-existing LAT nanoclusters in the plasma membrane [22,23]. At later points, LAT vesicles may facilitate TCR signaling amplifications, as observed in previous study in which ZAP70, and consequently membrane bound LAT, did not play a role in TCR phosphorylation [43]. Therefore, different spatial organizations of LAT may play different roles in the TCR signaling network [7,13,23,34,40]. Our findings highlight the importance of LAT clustering in early TCR signaling. Future studies could assess LAT cluster dynamics in T cells from immunodeficient or autoimmune patients to uncover potential diagnostic markers. Additionally, investigating whether disrupting LAT clustering alters T cell activation may inform new immunotherapy strategies. Studying LAT cluster formation in naïve, memory, and exhausted T cells could also reveal how LAT contributes to tuning TCR signaling outputs across different T cell states. These directions would help bridge our mechanistic insights with clinically relevant applications.

## 4. Materials and Methods

### 4.1. Star Methods

**Reagent or resource source identifier antibodies.**
Mouse anti-human CD3 (Clone OKT 3)eBioscience, Thermo Fisher Scientific (Waltham, MA, USA)Cat # 16-0037 RRID:AB_468855Mouse anti-human CD28 (Clone CD28.2)eBioscience, Thermo Fisher ScientificCat # 16-0289 RRID:AB_468926Mouse anti-human CD4 (Clone OKT4)Biolegend (San Diego, CA, USA)Cat # 317419 RRID: AB_571938Rabbit anti-human p44/42 MAPK (Erk 1/2) Cell Signaling (Danvers, MA, USA)Cat # 9102 RRID: AB_330744Rabbit anti-human phosphor p44/42 MAPK (Erk 1/2)Cell SignalingCat # 9101S RRID: AB_149900Rabbit anti-human LATCell SignalingCat # 9166S RRID: AB_10695247Rabbit anti-human phosphor-LAT (Tyr220)Cell SignalingCat # 3584S RRID: AB_2157728Goat anti-rabbit IgG (H + L)-HRPBio-Rad (Hercules, CA, USA)Cat # 1706515 RRID: AB_2617112Goat anti-mouse IgG (H + L)-HRPBio-RadCat # 1721011 RRID: AB_2617113Alexa Fluor 647-AffiniPure F(ab’)2 Fragment Goat Anti-rabbit IgG antibodyJackson ImmunoResearch (West Grove, PA, USA)Cat # 111-606-047 RRID: AB_2338082


**Bacterial strains.**
BL21(DE3) competent *E. coli*New England Biolabs (Ipswich, MA, USA)Cat # C2527HDH5-alpha competent *E. coli*New England BiolabsCat # C2987H


**Chemicals, peptides, and recombinant proteins.**
Poly-L-Lysine (PLL)Sigma-Aldrich (North Ryde, NSW, Australia)Cat # P8920Glucose oxidase type VIISigma-AldrichCat # G2133Horseradish peroxidase type VI-ASigma-AldrichCat # P6782HBSS, calcium, magnesiumThermo Fisher ScientificCat # 14025076Bovine serum albumins (BSA)Sigma-Aldrich Cat # A905616% paraformaldehyde (PFA)Thermo Fisher Scientific Cat # 28908BugBuster Merck Millipore (Bayswater, VIC, Australia)Cat # 70921ECL Western blotting substrateThermo Fisher ScientificCat # 32209ImidazoleSigma-AldrichCat # 15513Pre-casting 12% tris-glycine mini gels Thermo Fisher Scientific Cat # XP00120BOXIsopropyl β-D-1-thiogalactopyranoside (IPTG)Merck MilliporeCat # US1420322


**Critical commercial assays.**
Nickel-nitrilotriacetic acid (Ni-NTA) agaroseInvitrogen (Waltham, MA, USA)Cat # R90101Gold nanoparticle (100 nm)BBI Solutions (Crumlin, UK)Cat # EM. GC100No. 1.5 cover glassProSciTech (Kirwan, QLD, Australia)Cat # 0117580BCA protein assay kitThermo Fisher Scientific (Waltham, MA, USA)Cat # 23225


**Deposited Data.**

**Experimental models: Cell lines.**
Jurkat (Clone E6-1) cell lineATCC (Manassas, VA, USA)ATCC Cat # TIB-152, RRID CVCL_0367Jurkat LAT −/−This paperN/A


**Oligonucleotides.**
LATgRNA_P1S GCCCATCCTGGCCATGTTGAGTTTTAGAGCTAGIDTN/ALATgRNA_P1AS TCAACATGGCCAGGATGGGCCGGTGTTTCGTCCIDTN/ALATgRNA_P2S AGATCCCCGCAGCCCCTTGGGTTTTAGAGCTAGIDTN/ALATgRNA_P2AS CCAAGGGGCTGCGGGGATCTCGGTGTTTCGTCCIDTN/ALATgRNA_P3S GTGGCGAGCTACGAGAACGAGTTTTAGAGCTAGIDTN/ALATgRNA_P3AS TCGTTCTCGTAGCTCGCCACCGGTGTTTCGTCCIDTN/A


**Recombinant DNA.**
LAT-mEOS2(Williamson et al., 2011) [18]N/ALAT-mCherry(Williamson et al., 2011) [18]N/ALAT_YF_-mCherry(Williamson et al., 2011) [18]N/ALAT_YF_-mEOS2(Williamson et al., 2011) [18]N/ALATY132F-mCherryThis paperN/AZAP70-eGFPThis paperN/AGrb2-eGFPThis paperN/ACD4-LAT-mCherryThis paperN/AHis6-mcherryAddgeneAddgene Cat # 29722His6-eGFP

CRISPR/Cas9 expression vectorLaboratory of ZhengN/AgRNA expression vectorLaboratory of ZhengN/AGrb2-nostop cDNAAddgeneAddgene Cat # 70384


**Software and algorithms.**
Prism (v7)Graphpadhttps://www.graphpad.com/ (accessed on 22 March 2025)ImageJNIHhttps://imagej.nih.gov/ij/ (accessed on 22 March 2025)Matlab (2017a)MathWorkshttps://au.mathworks.com/ (accessed on 22 March 2025)Zen (2012)Zeisshttps://www.zeiss.com/ (accessed on 22 March 2025)CellProfilerCellProfilerhttp://cellprofiler.org/ (accessed on 22 March 2025)Density-based spatial clustering of application with noise (DBCAN)(Pageon et al., 2016) [33]N/A Degree of colocalization (DoC)(Pageon et al., 2016) [33]N/A k-Space imaging correlation spectroscopy (kICS)(Abu-Arish et al., 2015 [31]; Kolin et al., 2006 [32])N/ATemporal cross-correlation function (TCCF)(Sisan et al., 2010) [41]N/AStep fitting analysis (Cocucci et al., 2012) [30]N/A


**Contact for Reagent and Resource Sharing**

Further information and requests for reagents may be directed to Jieqiong Lou at jieqiong.lou@unimelb.edu.au

**Experimental Model**

Cell lines

Jurkat (Clone E6-1) and Jurkat LAT knock out cell lines were cultured at 37 °C in 5% CO_2_ in RPMI-1640 supplemented with 10% FBS and 2 mM L-glutamine.

Bacteria

*E. coli* BL21 (DE3) and DH5 alpha were cultured at 37 °C, with shaking at 220 rpm.

### 4.2. Method Details

#### 4.2.1. Cloning

Full length ORF of Grb2 and ZAP70 were cloned into peGFP-N1 using a standard digestion and ligation strategy. The extra-cellular domain of CD4 was cloned into pmCherry vector and followed by adding LAT transmembrane domain and cytosolic tail using a standard digestion and ligation strategy.

#### 4.2.2. Protein Expression and Purification

His-mCherry or His-eGFP were expressed in an *E. coli* (strain BL21(DE3)). The cells were cultured in LB broth at 37 °C until the optical density at 600 nm reached 0.6, after which protein expression was induced by 1 mg/mL IPTG. After induction, cells were further cultured for 3–4 h and harvested by centrifuge at 4500 rpm for 10 min. Cells were lysed by Bugbuster and the cell lysate was centrifuged at 15,000 for 15 min. His-mCherry in supernatant was purified by Ni-NTA resin according to the manufacturer’s instructions. Protein concentration was determined by BCA assay.

#### 4.2.3. Imaging Sample Preparation

Throughout the experiment, the cover glass was cleaned by rinsing with Milli-Q H_2_O, 100% acetone, ethanol, and dried by N_2_ flow. The cleaned cover glass was coated with poly-L-Lysine (PLL) by incubating with a 0.01% (*v*/*v*) PLL solution for 30 min at room temperature, removing the PLL solution and drying the cover glass for further 30 min, or with anti-CD3 and anti-CD28 by incubating with 10 µg/mL antibody solution for 1 h at room temperature. For SMLM samples, cells were attached to PLL (resting) or anti-CD3 and anti-CD28 (activated) coated cover glass for 2 or 10 min at 37 °C (specified in individual experiments). Resting and activated cells are fixed by 4% (*w*/*v*) paraformaldehyde (PFA) for 20 min at 37 °C. Fixed cells were directly used for imaging (photoactivated localization microscopy, PALM) or were further processed for immunofluorescence (IF) (directed stochastic optical reconstruction microscopy dSTORM). For IF, cells were permeablized by 0.1% Triton X-100, and blocked with 1% BSA, incubated with primary antibody at 4 °C for overnight and followed by fluorescent dye conjugated secondary antibody incubation for 1 h at room temperature. An oxygen-scavenging buffer (25 mM Hepes (PH 8.0), 25 mM glucose, 5% glycerol, 0.05 mg/mL glucose oxidase and 0.025 mg/mL horse radish peroxidase) was used for dSTORM imaging. For two-color SMLM imaging, 100 nm gold beads were added before imaging and allowed to settle for 20 min for image alignment. For testing actin polymerization in wild type and LAT knock out Jurkat cells, cells were fixed and F-actin was stained by incubating with Phalloidin-Alexa488 (1% *v*/*v*) for 30 min at room temperature. For CD4-LAT-mCherry live cell imaging, 5 µg/mL Alexa488 conjugated anti-CD4 antibody was incubated with 1 million CD4-LAT-mCherry transient transfected C3 cells for 30 min on ice. Cells were washed three times with cold PBS at 4 °C to remove excess antibody, and cells were re-suspended in 20 µL cold HBSS with calcium and magnesium buffer (Cat # 14025076, Thermo Fisher Scientific) after the last wash and incubated on ice before imaging to prevent endocytosis. For the rest of the live imaging samples (LAT-mCherry, LATYF-mCherry, LATY132F-mCherry, LAT-mEos2, LATYF-mEos2, LATY132F-mEos2, ZAP70-eGFP and Grb2-eGF), 1 million transient transfected C3 cells were centrifuged down and re-suspended in 400 µL HBSS at 37 °C. Half a million cells were used for each coverslip in live cell imaging experiments. For single fluorescent protein intensity quantification, 1.5 × 10^−12^ g/mL purified mCherry solution was incubated on cover glass for 10 min at room temperature and the cover glass was washed with PBS twice.

#### 4.2.4. Imaging

For all the following imaging experiments, the same total internal reflection fluorescence (TIRF) microscope (ELYRA, Zeiss (Jena, Germany)) and 100× oil-immersion objective lens (NA = 1.46) were used. Single snapshots for multiple fields of view were taken for actin polymerization in response to different conditions. For Alexa 488-CD4-LAT-mCherry live cell time-lapse movies, half a million transfected cells (10 µL) were added to coverslip with 200 µL pre-warmed HBSS imaging buffer, and for the rest of the proteins, half a million transfected cells (200 µL) were added to the dry coverslip. For SPT experiments, imaging acquisition started as soon as cells contacted the cover glass, which was defined as time 0, and one cell was imaged for each slide. LAT-mCherry was excited by a 561 nm laser (0.2%, 20 µW) and images were acquired by using a electron-multiplying charge-coupled device (EMCCD) camera (iXon DU-897, Andor (Belfast, UK)) with 150 gain and 200 ms exposure time (5 frame/s, 300 frames in total). For two-color movies, eGFP and mCherry were excited simultaneously by 488 (0.2%, 18 µW) and 561 nm (0.2%, 20 µW) lasers and images were acquired by two EMCCD cameras with the same gain and exposure time as for the one-color movie. Single mCherry images were taken by using same imaging profile as single color movies. For Alexa488-CD4-LAT-mCherry two-color movies, images were taken by using the same imaging profile as two-color SPT movies, except that they were imaged at 5 s/frame and 120 frames in total (10 min). For LAT-mEOS2 molecule diffusion, mEOS2 was photo-switched by (0.05%, 5 µW) 405 nm laser and imaged by 561 nm lasers (2% 1 mW). Images were acquired by the same EMCCD camera with 30 ms exposure (33.3 frame/s, 3000 frames in total) and 150 gain. Imaging began when cells started spreading on the cover-glass and 5 cells were imaged for each slide. For PALM and dSTORM two-color imaging, mEOS2 was photo-switched by 405 nm laser (0.1–0.3%, 10–40 µW)) and imaged by 561 nm laser (50%, 20 mW), and for Alexa 647 dSTORM, 633 nm laser (50%, 15 mW) illumination was used for imaging, with 405 nm laser (0.1–1%, 15 µW–1 mW) for conversion from the dark state. For PALM and dSTORM, 20,000 images were acquired per sample with an exposure time of 18 ms and gain of 150. For two-color acquisitions, the Alexa647 channel was acquired first, followed by mEOS2. Raw fluorescence intensity images were analyzed with Zen 2012 software, generating tables containing the x-y particle coordinates of individual localization with 25.6 nm mean localization precision (Figure S4A) detected in the acquisition. Multiple localizations that appeared in a PSF width (100 nm) within 50 frames (off-gap) were summed and counted as one molecule.

#### 4.2.5. Imaging Analysis

##### Cluster Analysis of One-Color SMLM Data

DBSCAN analysis [34] was used to identify individual clusters. The DBSCAN method detects clusters using a propagative method that links points belonging to the same cluster based on two parameters: the minimum number of neighbors Ɛ (Ɛ = 3) in the radius of r (r = 20 nm), and clusters containing at least 10 molecules. The DBSCAN routine was implemented in MATLAB and subsequently coded in C++ and compiled in a MEX file.

##### Degree of Colocalization (DoC) Analysis of Two-Color SMLM Data

For detailed information on DoC analysis please refer to [33]. Briefly, first, molecules with local density below a random distribution, which equals the total number of molecules divide by the total area of the region of interest, were excluded to reduce the size of the data set. Then for each molecule, the local density of each channel is calculated at increasing radius sizes (10–500 nm), providing the density gradient around that molecule for each channel. The two density gradients are tested for correlation. As a result, each molecule is scored with a DoC based on correlation, with DoC ranging from −1 to 1, where1 characterizes colocalization, 0 corresponds to random distribution and −1 defines segregation. Molecules with a DoC score ≥ 0.4 were defined as colocalized molecules based on simulation results [33]. In the next step, individual clusters were detected using a DBSCAN analysis, similar to the one-color analysis, and the clusters were subsequently separated into colocalized clusters (containing more than 10 molecules with DoC score above 0.4) and non-colocalized clusters. These two populations of clusters were then analyzed to extract size, circularity, density and mean DoC score, and any other type of information. From the local DoC score of each molecule, taken at a radius of 20 nm and normalized to the average DoC of the region, a pseudo map is created in which each molecule is represented with a color code corresponding to the mean DoC value.

##### Cluster Tracking, Single mCherry Intensity Quantification and Step Fitting Analysis

LAT clusters in live cell TIRF movies were tracked in CellProfiler. Step 1, identify cells: images were smoothed with a median filter 30 pixel in diameter and cells were identified based on the adaptive threshold strategy and Otsu thresholding method. Then cell outlines were expanded for 8 pixels. Step 2, identify LAT clusters: the raw images were fine smoothed with a Gaussian filter 2 pixel in diameter, speckle features in fine smoothed images were enhanced at a defined diameter of 6 pixels, and enhanced images were masked by cell outlines identified in step 1. LAT clusters (objects with a diameter of 6 pixels) were identified based on a per object threshold strategy and the robust background thresholding method. Overlapped LAT clusters were distinguished based on intensity and dividing lines between overlapped clusters were drawn based on the object’s intensity profile. Step 3, track LAT clusters: identified LAT clusters were tracked based on the overlap method since nascent plasma membrane LAT clusters were very static in position, with the maximum pixel distance to consider matches being 3 pixels for clusters in frame to frame. The minimum trajectory lifetime is set to 1 in order to include all the potential trajectories. Clusters satisfying the following conditions—lifetime ≥ 30 frames (6 s), trajectory is not detected in frame 1, and diffusion coefficient is smaller than 0.05 μm^2^/s—were selected for subsequent step fitting analysis. In order to detect the initial phase of LAT cluster assembly, the mean intensity (same coordinate of LAT cluster in the first detected frame, and 6 pixel in diameter) of 20 frames in advance of the first detected frame was added for the individual trajectory. For two-color live cell movies, for ZAP70 and LAT, the ZAP70 cluster was tracked according to the same single color tracking algorithm and LAT cluster intensity in the corresponding frame and position was measured. For LAT and Grb2, the LAT cluster was tracked and Grb2 cluster intensity in the corresponding frame and position was measured.

Single mCherry was identified by Gaussian fitting at 6 pixels in diameter and a signal-to-noise ratio above 6 in ImageJ. The intensity of the same coordinate was measured in the entire movie and plotted. Single mCherry intensity was calculated as the mean intensity difference before and after single step photobleaching. The mean intensity and standard deviation were used for the following step fitting analysis.

##### LAT Vesicles Number Quantification

##### Step Fitting Analysis of Fluorescence Intensity Traces

Step fitting was carried out using an algorithm implemented in Matlab (Mathworks, Natick, MA, USA) as described previously [45]. The algorithm fits steps of equal intensity to an individual intensity trace, whereby missing steps (arrival of more than one molecule from one frame to the next) are allowed. The step size can vary between intensity traces, and is determined for each trace on the basis of the mean and standard deviation of the intensity of the fluorescent protein determined from a calibration experiment. The calibration experiments were carried out by recording TIRF movies of purified free mCherry adsorbed onto coverslips.

##### kICS Confinement Analysis of LAT in Live Cells

In order to quantify the diffusion of LAT molecules and their confinement by docking clusters, we applied k-space image correlation spectroscopy (kICS) confinement analysis, implemented in Matlab (Mathworks, Natick, MA, USA) as previously reported [31,32,35,36,37]. Briefly, we took an image time series of LAT-mEOS2, imaged at 33.3 Hz, in either resting or activated T-cells and calculated the zero-temporal lag normalized kICS correlation function), in Fourier (k-space) as described previously [31,32,35,36,37]. Next, we circularly averaged the correlation function, assuming isotropic diffusion, at each temporal lag tau, which averages out the noise and amplifies the constructive correlations for a given spatial frequency k^2 (Figure S3A). The resulting correlation function evolves in spatial frequency k^2 and temporal lags tau. In the following step, we fitted the resulting correlation function vs. the spatial frequency, at a given temporal lag, with the sum of exponential decays (Figure S3B). Out of this fit, we obtained two amplitudes and two decay rates as a function of temporal lag. One amplitude and decay rate are related to large spatial scale movement and fast diffusion of LAT molecules outside of clusters, referred to in references [31,35,36,37] as the free component. The other amplitude and decay rate recovered are related to the small spatial scale movement of LAT molecules within and around clusters, referred to in references [31,35,36,37] as the confined component. We characterized the amplitudes and decay rates by fitting linearly defined ranges of temporal lags (tau) to recover several confinement parameters used for comparison between experimental conditions studies in this experiment. First, three temporal lags of either free or confined decay rates were fitted vs. temporal lag, to recover the diffusion coefficients of LAT molecules in large spatial scales outside clusters (Dfree) and inside clusters (Dconfined), respectively (Figure S3C,D). The average decay rate for the micro component at late temporal lags was used to calculate the confinement diameter of LAT molecules at small spatial scales (Figure S3D). This parameter, labelled as confinement, has been shown [31,35,36,37] to be directly proportional to the actual size of confining clusters, but also inversely proportional to the confinement strength (measure of probability for keeping LAT molecules inside the clusters). Finally, the ratio of amplitude of the macro component to that of the micro component is directly proportional to the density of molecules outside vs. molecules inside of clusters (Figure S3E) [31,35,36,37]. Late temporal lag value of this ratio was used as the extracted confinement parameter, since the correlation function fully collapses into two dynamical populations for those tau values.

##### Event Ordering in LAT Clusters

In our two-color experiments, with either LAT-Grb2 or LAT-ZAP70 interacting pairs, we assessed the order of binding of these molecules to co-clusters. We quantify the extracted cluster intensity evolving in time, for both proteins (channels), by calculating the asymmetries in the temporal cross-correlation function (TCCF), similar to the work reported in reference [41]. For intensities traces for a pair of proteins, recovered from a given cluster, we calculated the temporal cross-correlation function where a protein 1 intensity, for example LAT in the LAT-Grb2 pair, is a reference signal, while the other protein intensity is being shifted. We call this correlation function LAT to Grb2 (or forward TCCF). Then we perform the same calculation with the protein 2 intensity as a reference signal and protein 1 intensity as a shifting signal. We call this function Grb2 to LAT (or backward TCCF). Finally, we compute the difference of the forward and backward TCCFs, deltaCC = (LAT to Grb2)—(Grb2 to LAT), which evolves over temporal lags tau. As shown in [41] deltaCC will be zero for all lags if protein 1 binds to a cluster independent of protein 2 being bound or not. On the other hand, deltaCC will be positive if protein 2 binds, for some average time, after protein 1 was bound to the cluster. Similarly, deltaCC will be negative if protein 2 binds, for some average time, before protein 1 binds the cluster. Sisan and colleagues [41] characterized even further deltaCC and extracted the characteristic average time protein 1 is bound to the cluster prior to binding of protein 2, but in this study, data exhibited several binding–unbinding cycles per cluster, making the model proposed more valid for analysis. Our data show a single rise in cluster intensity, in either protein 1 or 2 channels, and as such, deltaCC is not sufficiently defined to recover quantitative information on average binding–unbinding times. In order to assess the binding ordering of LAT vs. Grb2 or ZAP70, we calculate the cumulative sum of deltaCC up to a temporal lag of 50 frames. If the cumulative sum of deltaCC is positive, than protein 1 is leading protein 2, while a negative value indicates that protein 1 lags behind protein 2. Temporal cross-correlation functions were calculated in Matlab (Mathworks, Natick, MA, USA) by custom written software. The intensity traces were normalized to their time point 1, so that both proteins signals start at value 1, ensuring that the absolute values of intensity (which depend on fluorophores used, laser intensities, etc.) were not weighing one signal over another.

##### Statistical Analysis

All statistical analysis was performed using GraphPad software (Prism 10). Statistical significance between datasets was determined by performing two-tailed Student’s *t* tests. Graphs show mean values, and error bars represent the SD. In statistical analysis, *p* > 0.05 is indicated as not significant (n.s.), whereas statistically significant values are indicated by asterisks as follows: * *p* ≤ 0.05, ** *p* < 0.01, *** *p* < 0.001 and **** *p* < 0.0001.

## Data Availability

The microscopy data presented in this study are available upon request from the corresponding author due to the large size of single-molecule localization microscopy and single-particle tracking data.

## Supplementary Materials

The following supporting information can be downloaded at: https://www.mdpi.com/article/10.3390/ijms26094076/s1.
