# Supplementary material for: Step-Wise Assembly of LAT Signaling Clusters Immediately After T Cell Receptor Triggering Contributes to Signal Propagation
Source: Int J Mol Sci. 2025 Apr 25;26(9):4076. doi: 10.3390/ijms26094076 (PMC12071625; doi:10.3390/ijms26094076)
Supplement: Supplementary file 1 [file ijms-26-04076-s001.zip › ijms-3571674-supplementary-xml.pdf]

Supplementary Material

Step-wise assembly of LAT signaling clusters immediately after T cell receptor triggering contributes to signal propagation

Jieqiong Lou[1,2,3*], Elvis Pandzic[4*], Till Böcking[1,2], Qiji Deng [1,2], Jérémie Rossy[1,5], Katharina Gaus[1,2]

1. EMBL Australia Node in Single Molecule Science, School of Biomedical Sciences, University of New South Wales, Sydney, NSW 2052 Australia; till.boecking@unsw.edu.au (T.B.); qiji.deng@petermac.org (Q.D.);
jeremie.rossy@bitg.ch (J.R.); k.gaus@unsw.edu.au (K.G.)
2 ARC Centre of Excellence in Advanced Molecular Imaging, University of New South Wales, Sydney, NSW 2052, Australia
3 School of Physics, University of Melbourne, Melbourne, VIC 3010, Australia
4 Katharina Gaus Light Microscopy Facility, Mark Wainwright Analytical Centre, University of New South Wales, Sydney, NSW 2052, Australia
5 Biotechnology Institute Thurgau, University of Konstanz, 78464 Konstanz, Germany
* Correspondence: jieqiong.lou@unimelb.edu.au (J.L.); e.pandzic@unsw.edu.au (E.P.)

Movie 1. CD4-LAT-mCherry that contains the extracellular domain of CD4 on the N-terminal of LAT and mCherry on the C-terminal of LAT was expressed in LAT-deficient Jurkat cells and live cells stained with anti-CD4 antibodies conjugated to AlexaFluoro488 (green). Thus, LAT in the plasma membrane appears yellow and intracellular LAT appears red in merged TIRF images. Cell was activated by anti-CD3 and anti-CD28 antibodies coated surfaces and imaging started assoon as cell contacted with cover slips and imaged at 5 s/frame for 10 min at 26 °C.

Movie 2. LAT-deficient Jurkat cells expressing LAT-mCherry were exposed to non-activating surfaces. TIRF images starts as soonas cell was found in TIRF field and was imaged at 5 frames/s rate for 300 frames at 26 °C.

Movie 3. LAT-deficient Jurkat cells expressing LAT-mCherry were exposed to activating surfaces (anti-CD3 and anti-CD28 antibodies coated coverslips, Activating). TIRF images starts as soon as cell was found in TIRF field and was imaged at 5 frames/s rate for 300 frames at 26 °C.

Movie 4. LAT-deficient Jurkat cells expressing LAT-mEOS2 were exposed to non-activating surfaces (poly-L-lysine coated coverslips, Resting, Left) and surfaces activating (anti-CD3 and anti-CD28 antibodies coated coverslips, Activating, Right) for 0-5 min. Small portion of mEOS2 was photo-converted by 405 nm laser. Cells were imaged by 561 nm laser at 33 frames/s rate for 3000 frames at 26 °C.

Movie 5. LAT-deficient Jurkat cells expressing LAT_{YF}-mCherry were exposed to activating surfaces (anti-CD3 and anti-CD28 antibodies coated coverslips, Activating). TIRF images starts as soon as cell was found in TIRF field and was imaged at 5 frames/s rate for 300 frames at 26 °C.

Movie 6. LAT-deficient Jurkat cells expressing LAT_{YF}-mEOS2 were exposed to non-activating surfaces (poly-L-lysine coated coverslips, Resting, Left) and surfaces activating (anti-CD3 and anti-CD28 antibodies coated coverslips, Activating, Right) for 0-5 min. Small portion of mEOS2 was photo-converted by 405 nm laser. Cells were imaged by 561 nm laser at 33 frames/s rate for 3000 frames at 26 °C.

Movie 7. LAT-deficient Jurkat cells expressing ZAP70-GFP (green) and LAT-mCherry (red) were exposed activating surfaces (anti-CD3 and anti-CD28 antibodies coated coverslips). TIRF images starts as soon as cell was found in TIRF field and was imaged at 5 frames/s rate for 250 frames at 26 °C.

Movie 8. LAT-deficient Jurkat cells expressing Grb2-GFP (green) and LAT-mCherry (red) were exposed activating surfaces (anti-CD3 and anti-CD28 antibodies coated coverslips). TIRF images starts as soon as cell was found in TIRF field and was imaged at 5 frames/s rate for 300 frames at 26 °C.

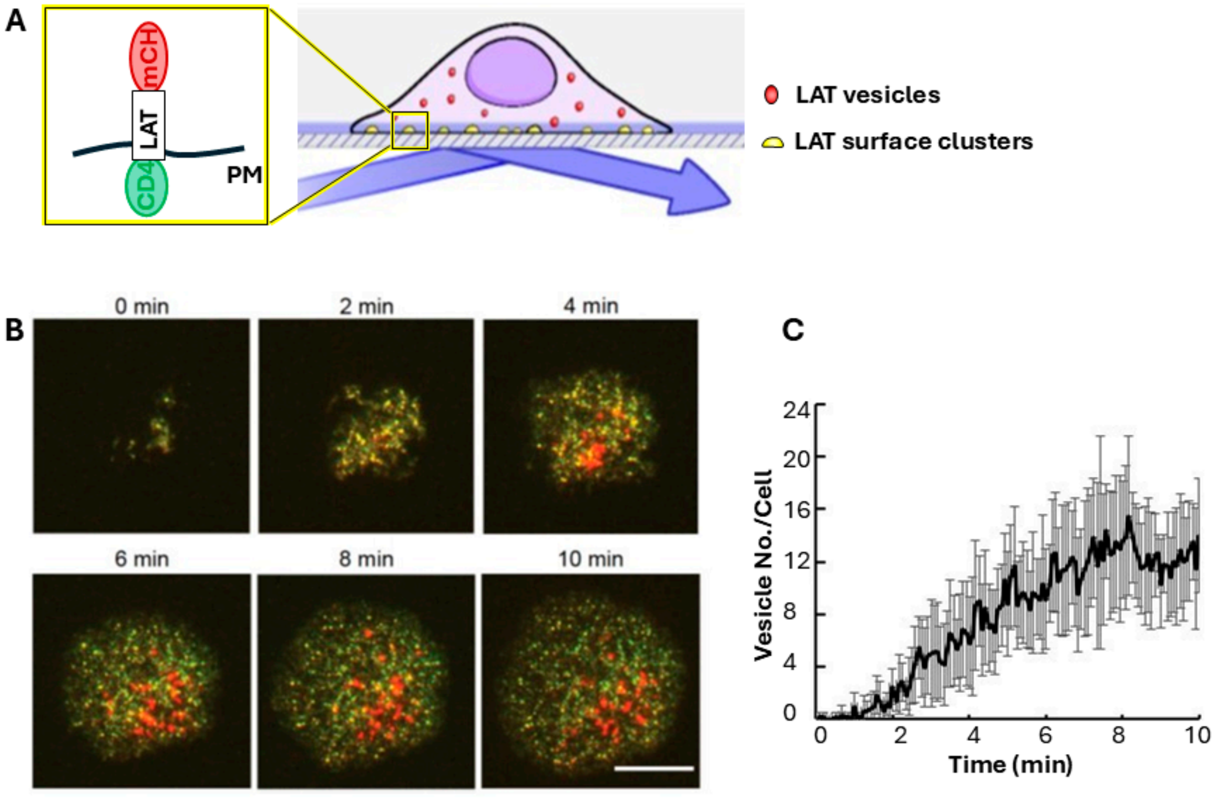

Figure S1. Time post TCR triggering distinguishes LAT in the plasma membrane from vesicular LAT.

(A) Schematic of LAT surface cluster and vesicle tracking experiment. Cells are pre-labelled with cell-impermeable anti-CD4 antibodies conjugated to Alexa488 on ice so that surface LAT appears yellow and vesicular LAT red. T cells are activated on antibody-coated coverslips with both LAT vesicles and LAT cell surface clusters located within the TIRF zone.
(B) Live-cell TIRF images of CD4-LAT-mCherry expressed in LAT-deficient Jurkat cells on anti-CD3 and anti-CD28 antibody-coated coverslips for 10 min. Images are representative of five independent experiments. Scale bar =10 μm.
(C) Quantification of cell surface and vesicle LAT in the first 10 min upon anti-CD3 and anti-CD28 stimulation (Error bar, standard deviation, results are average of 6 cells in three independent experiments).

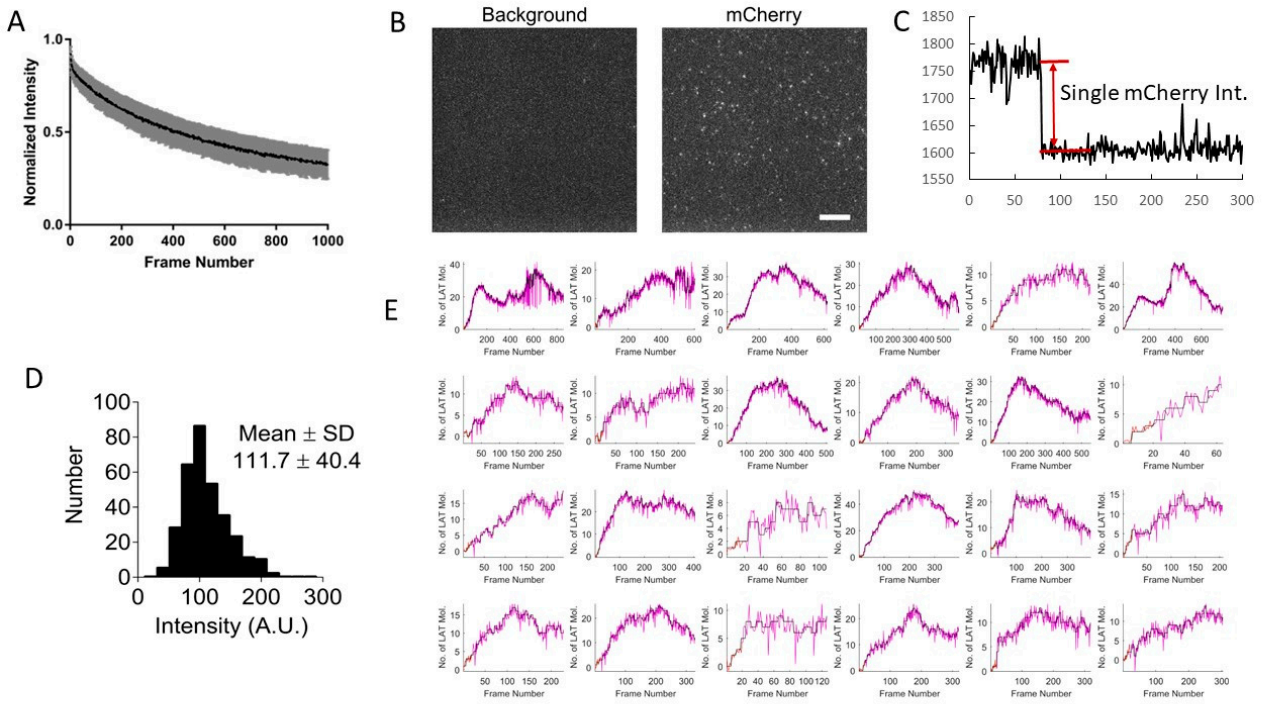

Figure S2. Related to Figure 2. Imaging of individual mCherry molecules (A-C) and SPT tracking of individual LAT-mCherry assemblies (D).
To ensure that individual LAT-mCherry molecules could be detected and tracked with TIRF microscopy, the intensity of individual mCherry molecules expressed and purified from E. Coli was determined under the identical TIRF imaging conditions as data shown in Figure 1.
(A) Photobleaching quantification. LAT-mCherry intensity over time in fixed Jurkat cell under the same imaging condition as live cell movies (Error bar, standard deviation, n=10)
(B) Images of glass coverslips without (background) and with purified mCherry molecules (mCherry). Scale bar = 5 μm.
(C) Example of single step photobleaching curve of purified single mCherry
(D) Distribution of mCherry intensity was fitted to a Gaussian distribution centered at 111.7 AU with a width of 40.4 AU.
(E) Examples of individual LAT assemblies (pink) fitted to the step-fitting algorithm (purple) to yield the number of LAT molecules.

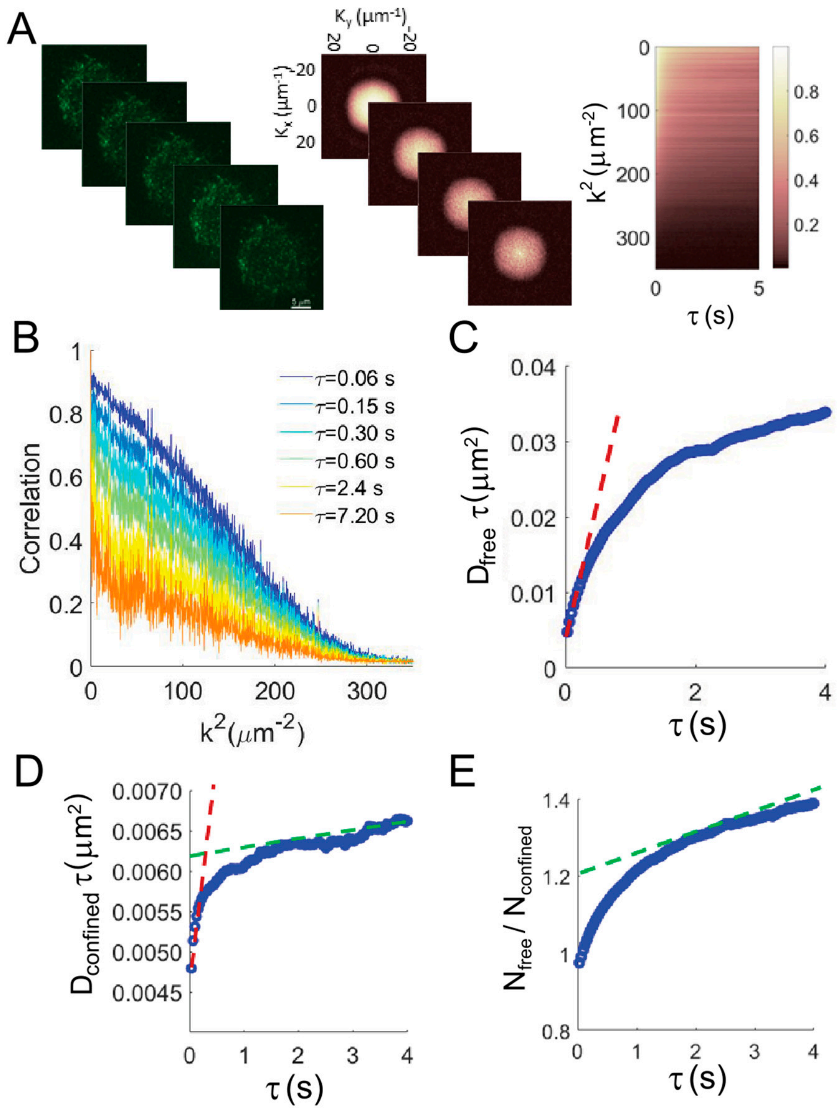

Figure S3. Related to Figure 2. kICS analysis of LAT mobility from TIRF image time series.
(A) Examples from the TIRF intensity image time series of WT LAT-mEos2 in activating T cells (A, left). Images were 2D (spatially) Fourier transformed and temporally correlated (A, middle), resulting in correlation functions that were azimuthally averaged (for isotropic diffusion) and normalized to zero temporal lag ($\tau = 0$) to remove contribution from the point spread function (A, right). Scale bar = 5 μm,
(B) Correlation function decay curves as a function of spatial frequencies squared (k^2) are a sum of two exponentials at every temporal lag (τ). Blue to orange traces indicate early to late temporal lags, respectively.
(C-D) Fitting the correlation functions, shown in B, to two exponential decays at every tau yields two curves of $D * \tau$ *versus* τ for two different length scales, where D is the diffusion coefficients. We named the LAT population diffusing in the larger scale "free" (C) and the population diffusing on the shorter scale "confined" (D). Fitting the first five temporal lags (red dotted line) gives the diffusion coefficient, D_{free} and $D_{confined}$, while the intercept of the plateau of confined population (green dotted line in D) gives the length scale on which this population of LAT molecules experienced confinement.
(E) The ratio of amplitudes of free to confined components at late temporal lags (dashed green line), $N_{free}/N_{confined}$, is proportional to the ratio of densities of molecules that probe the large spatial scale "free" *versus* molecules diffusing on the shorter scale spatial scale "confined".

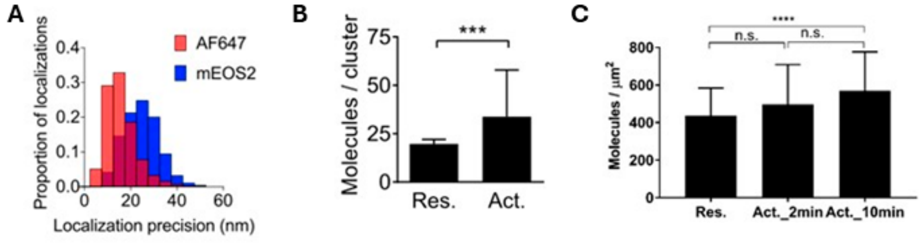

Figure S4. Related to Figure 3. SMLM imaging and cluster analysis
Experimental conditions were as described for Figure 3.
(A) Histograms of localization precision for LAT-mEOS2 and phosphorylated LAT (pLAT) probed with Alexa Fluor647-conjugated antibodies.
(B) Number of LAT molecules per cluster in resting (Res.) and 2 min post activation with antibody-coated coverslips (Act).
(C) Number of LAT molecules per membrane area in resting T cells (Res.) and in T cells activated (Act.) on antibody-coated coverslips for 2 min and 10 min. Data in B-C are mean and standard deviation of ≥ 30 ROI per condition. n.s. *P*>0.05 *** *P*<0.001, **** *P*<0.0001, (unpaired t test).

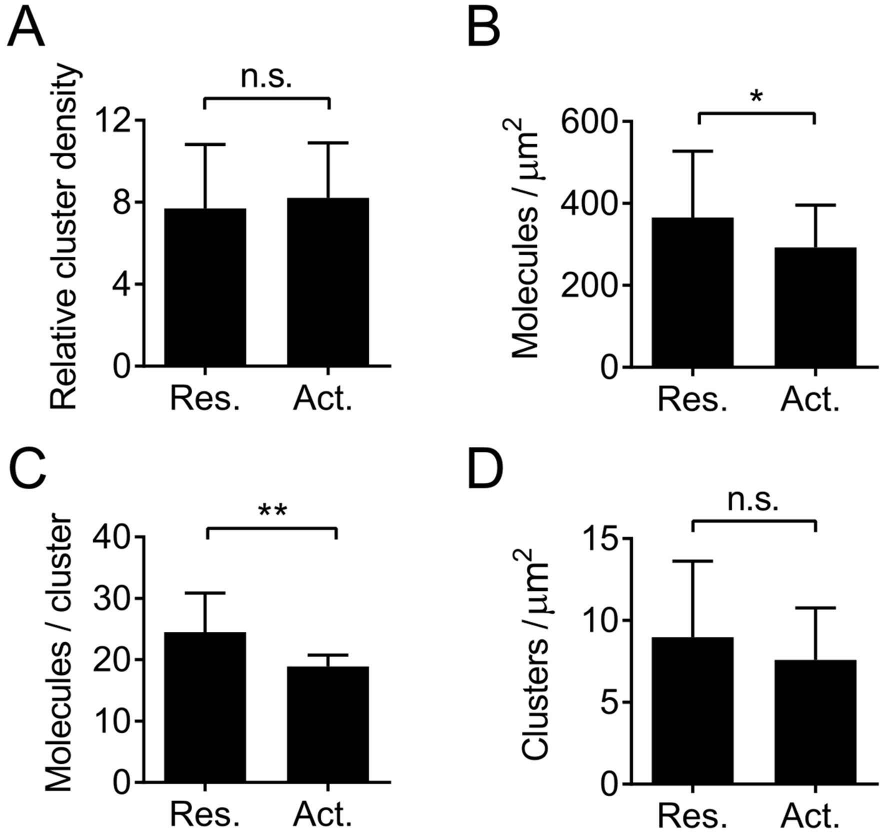

(

Figure S5. Related to Figure 4. LAT_{YF} cluster characteristics in resting and activating cells.
Experimental conditions were as described for Figure 4.
(A-D) Relative density of LAT_{YF} molecules in clusters (A), number of LAT_{YF} molecules per area (B), number of LAT_{YF} molecules per cluster (C) and number of LAT_{YF} clusters per area (D) in resting (Res.) and activating T cells (Act.) 2 min post stimulation. Data are means and standard deviations of ≥ 10 cells per condition. ns, not significant, * $P<0.05$, ** $P<0.01$ (unpaired t test).

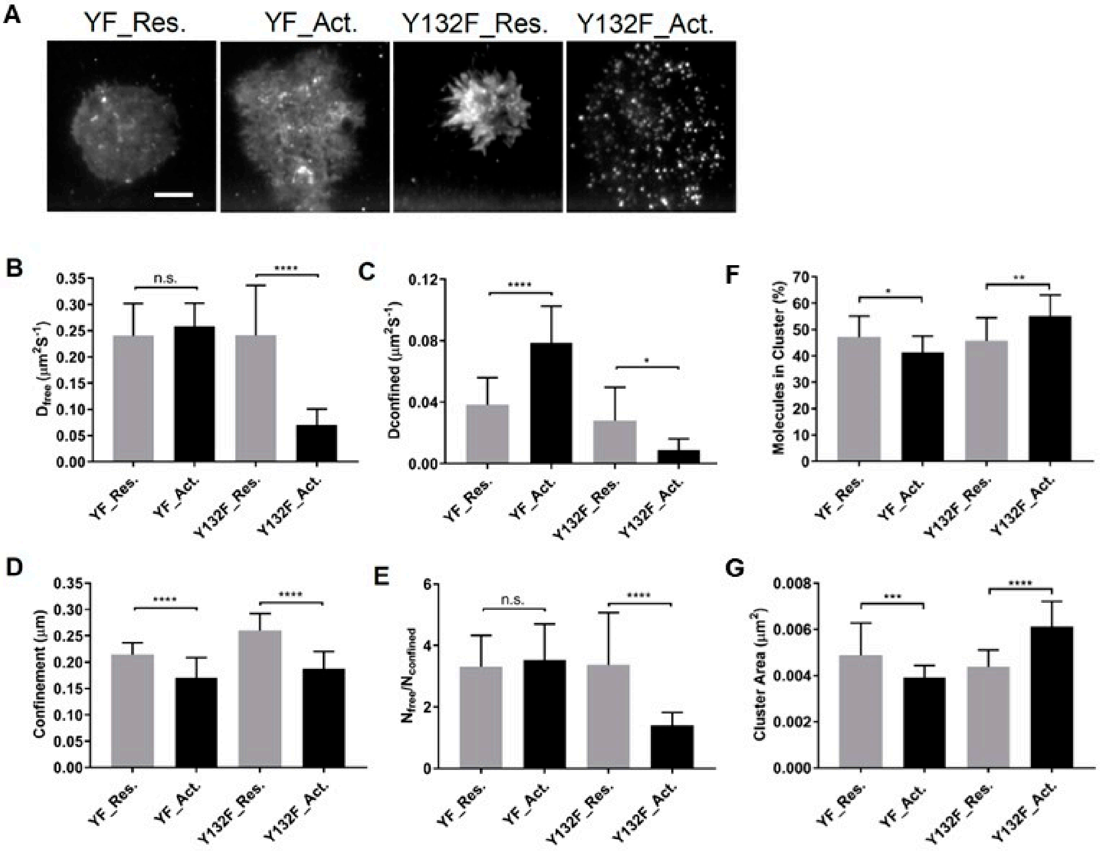

Figure S6. Related to Figure 4. **LAT_{Y132F} cluster characteristics in resting and activating cells.** Experimental conditions were as described for Figure 4.

A.Merged TIRF images of 3,000 frames (mean intensity) of LAT_{YF}-mEOS2 (Left) or LAT_{Y132F}-mEOS2 in a resting T cell (YF_Res. or Y132F_Res.) and activating T cells (YF_Act. or Y132F_Act.) . Scale bar = 5 µm.

B-E. KICS analysis quantify the diffusion coefficients of the 'free' (B) and 'confined' (C) populations, the length scale of the 'confined' population (D), and the ratio of LAT molecules in the 'free' *versus* 'confined' population (E) (n ≥8 cells ROIs from 2 independent experiments)

F-G. SMLM imaging of LAT_{YF}-mEOS2 or LAT_{Y132F}-mEOS2 quantify the of percentage of molecule in cluster (F) and cluster area (G) in resting and activating LAT-deficient Jurkat cells (n ≥30 ROIs from at least 7 cells at 2 independent experiments). In B-G data are means and standard deviations. n.s. $P>0.05$, * $P<0.05$, ** $P<0.01$, *** $P<0.001$, **** $P<0.0001$ (unpaired t-test).
